# Effects of Forging and Heat Treatment on Martensite Lath, Recrystallization and Mechanical Properties Evolution of 18Ni(250) Maraging Steel

**DOI:** 10.3390/ma15134600

**Published:** 2022-06-30

**Authors:** Shucong Xu, Lin Yuan, Wenchen Xu, Debin Shan, Bin Guo

**Affiliations:** 1 National Key Laboratory for Precision Hot Processing of Metals, Harbin 150001, China; xushucong1995@163.com (S.X.); shandebin@hit.edu.cn (D.S.); guobin@hit.edu.cn (B.G.); 2 School of Materials Science and Engineering, Harbin Institute of Technology, No. 92 Xi Dazhi Street, Harbin 150001, China

**Keywords:** maraging steel, forging, heat treatment, microstructural and mechanical evolution, strengthening mechanism

## Abstract

The manufacturing process of maraging steel parts include forging, heat treatment and other technological links, and the strengthening mechanism at different stages is different, which has an important impact on the process design of forgings. To investigate the strengthening behavior of maraging steel, forging experiments with different deformation amounts and heat treatment conditions were carried out, and the microstructural and mechanical properties evolution of 18Ni(250) steel was analyzed. The experimental results show that the size of the martensite lath is affected by multiple factors such as the influence of grain size, recrystallization and martensite substructure fraction. The strengthening mechanism of maraging steel during forging and heat treatment is different. Forging combined with heat treatment can refine grains, and the internal defects of the original material can be better eliminated. The thermal deformation can better play the role of grain refinement compared with cyclic phase transformation, which can improve the plasticity of 18Ni(250) maraging steel.

## 1. Introduction

Maraging steel is a kind of ultra-high strength steel, with its advantages of high strength and toughness, low hardening index, good formability, simple heat treatment process and good welding performance. Maraging steel has become an indispensable material in high-tech areas such as rocket engine shell, the main shaft of aircraft engines, submarine shells and cryogenic missiles [1,2,3,4]. Different from traditional high strength steel, maraging steel is strengthened by the dispersion precipitation of intermetallic compounds instead of carbon, and fine intermetallic particles such as Ni_3_(Ti, Mo), Fe_2_Mo and Fe_7_Mo_6_ are precipitated during solution treatment at 810–860 °C and an aging treatment at 450–550 °C [5,6,7,8]. Common maraging steels include three typical series:18Ni, 20Ni and 25Ni, of which the 18Ni series is the most widely used [9,10,11,12,13,14].

In recent decades, the microstructural and mechanical evolution and strengthening behavior of maraging steel have been extensively studied. Aging treatment promotes a drastic increase in strength along with a drop in tensile ductility. Bai YC et al. found that solution treatment (ST) and solution + aging treatment (SAT) have a significant effect on the microstructure evolution. ST reduces the microhardness and tensile strength. SAT can improve the microhardness and tensile strength significantly, and both fracture elongation and impact toughness are reduced [15]. The microstructure of maraging steel is mainly lath martensite and metal compound precipitation, and the changes in tensile strength and ductility are mainly affected by the strengthening precipitates and microstructure defects [16,17]. In addition, with the increase of ageing temperature, the spacing of precipitated phases would increase, leading to the improvement of cracking resistances of maraging steel [18]. The effect of aging time on the microstructure and mechanical properties of maraging steel was investigated by Zhu HM et al. Because of the combined contributions of precipitation strengthening and softening induced by formation of reverted austenite, the microhardness and tensile strength is first increased and then decreased [19]. Under over ageing conditions, the optimum microstructure of maraging steel is comprised of martensite with a large amount of metal compound precipitation and reverted austenite [20,21,22,23]. The reverted austenite exhibited the TRIP effect, which make maraging steel obtain a combination of high strength and ductility [24,25,26,27,28,29,30,31]. The influences of cyclic transformation α→γ on the grain size of the maraging steel was studied by many scholars. Cyclic phase transformation can significantly refine the grain size, which can improve the plasticity of maraging steel [32,33,34]. The research of maraging steel mainly focuses on the heat treatment, cyclic phase transformation as well as the types and functions of precipitated phases. But there are few studies on the microstructural and mechanical evolution and strengthening mechanism of maraging steel during forging deformation. The hot working behavior of maraging steel was investigated, and the optimized hot processing window was obtained [35,36,37,38]. The hot compression deformation behavior of 18Ni maraging steel was investigated by Ren YH et al., and the complete recrystallization region was obtained [39].

The manufacturing process of maraging steel parts include forging, heat treatment and other technological links, and the strengthening mechanism at different stages is different, which has an important impact on the process design of forgings. For example, the main shaft forgings of aircraft engines of 18Ni(250) steel have high performance requirements, such as no microstructure defects and a tensile strength of 1800 MPa. However the shaft forgings have problems such as substandard grain size, unstable performance and poor consistency, which limit the further development of robot intelligent forging. The research on the strengthening mechanism and microstructural and the mechanical evolution of maraging steel is helpful for the design of the robot free forging process, which can improve the intelligent manufacturing level of aviation key forgings of maraging steel. However, the current research on maraging steel after forging has only obtained the constitutive relation and the processing map, and has not analyzed the strengthening mechanism during forging deformation. Therefore, in order to improve the final properties of 18Ni(250) maraging steel forgings, the research on microstructural and mechanical evolution and strengthening mechanism of 18Ni(250) maraging steel under different forging processes has important practical significance.

This paper studies the effects of different forging processes and heat treatment conditions on the microstructural and mechanical evolution of 18Ni(250) maraging steel, and explores the main strengthening mechanism during forging and heat treatment, which provides a reference for the intelligent forging process design of the aviation key forgings.

## 2. Experiments

### 2.1. Thermal Compression Experiment of Small Samples

**Figure 1:**
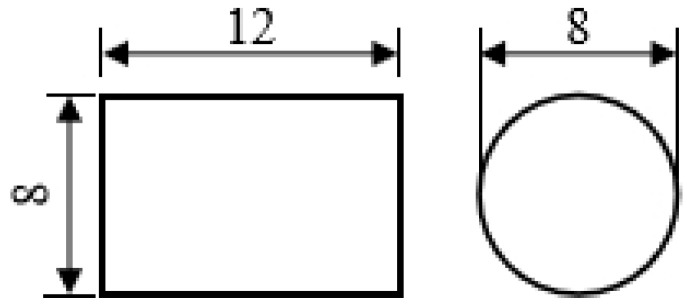
Thermal compression sample geometry (lengths in mm).

The thermal compression experiment was performed on samples with 8 mm in diameter and 12 mm in thickness. The samples were heated to the specified temperature at a rate of 10 °C/s and heat preserved for 20 min. Upsetting the samples to the specified deformation amount with a Gleeble 1500 thermal simulation machine. After upsetting, quenching the samples immediately in cold water to retain the microstructure. The shape of the thermal compression sample is shown in Figure 1.

### 2.2. Upsetting Experiment of Large Samples

**Figure 2:**
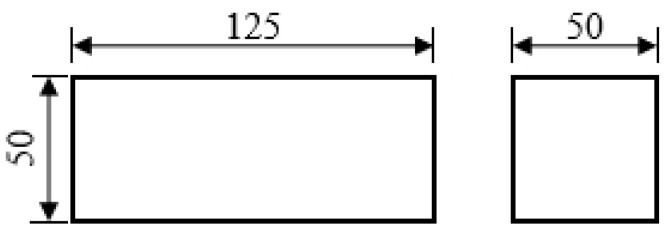
Upsetting sample geometry (lengths in mm).

The upsetting deformation experiment was performed on the samples with a size of 125 mm × 50 mm × 50 mm under the pneumatic hammer. The samples were heated to the specified temperature at a rate of 15 °C/min and heat preserved for 1 h. Upsetting the samples to the specified deformation amount. Then the compressed samples were air cooled, which is convenient for subsequent mechanical property testing. The shape of the upsetting sample is shown in Figure 2.

### 2.3. Heat Treatment Experiment

**Figure 3:**
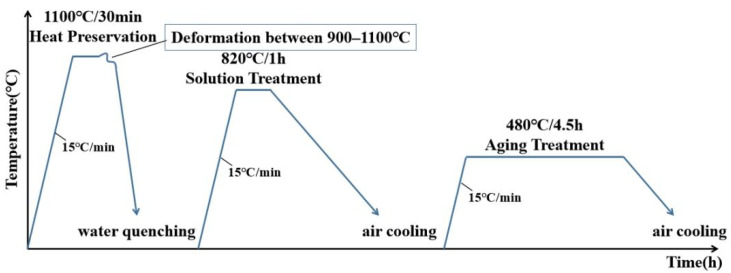
Schematic of the heat treatment.

A two-step heat treatment was carried out on the samples after compression experiment or upsetting experiment, the heating rate was set as 15 °C/min. Samples were austenitised at 820 °C for 1h and tempered at 480 °C for 4.5 h. The samples were then air cooled. A schematic diagram showing the overall process is given in Figure 3.

### 2.4. Tensile Experiment

**Figure 4:**
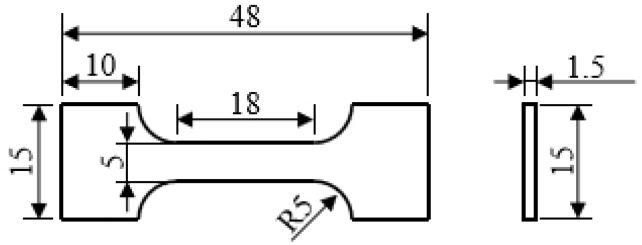
Tensile sample geometry (lengths in mm).

The tensile experiments were carried out under the electronic universal testing machine AG-X Plus. The tensile samples were taken from the core of the compression samples in the compression direction and perpendicular to the compression direction respectively. The shape of the tensile sample is shown in Figure 4. Tensile tests were conducted three times for each condition.

## 3. Results

### 3.1. The Morphology of Thermal Compression Samples

**Figure 5:**
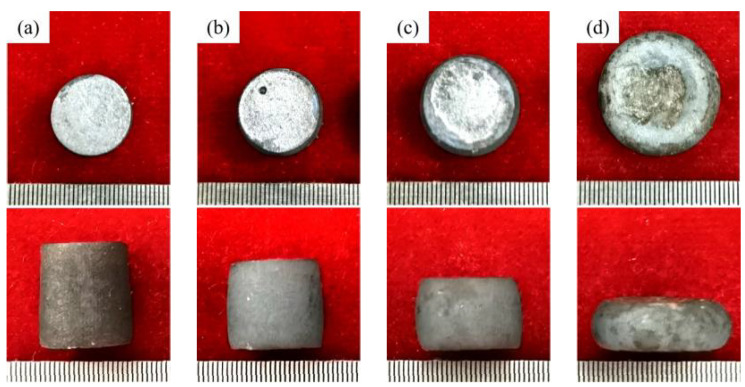
The morphology of the thermal compression samples: (**a**) 15%; (**b**) 30%; (**c**) 45%; (**d**) 60%.

The thermal compression experiment of 18Ni(250) maraging steel was carried out. The tests were carried out at the temperature of 1000 °C, a strain rate of 10 s^−1^, and the deformation amounts were 15%, 30%, 45% and 60%, respectively. The experiment results are shown in Figure 5. After the plastic deformation of maraging steel, the deformation is relatively uniform, and there are no cracks on the surface. The maraging steel samples have favorable plastic deformation ability.

### 3.2. The Morphology of Upsetting Samples

**Figure 6:**
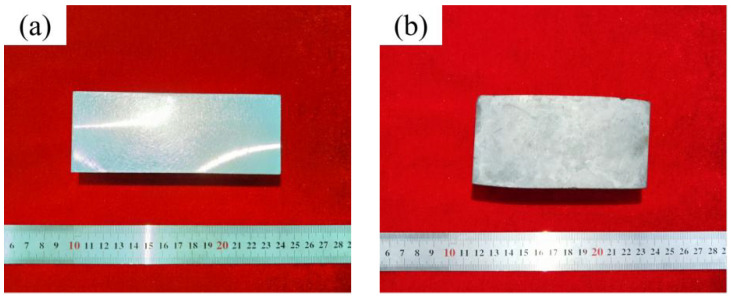

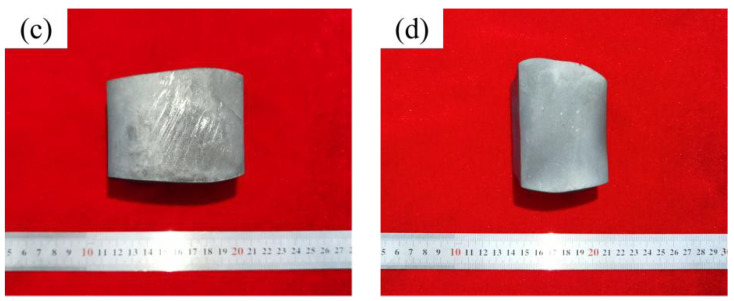
The morphology of the upsetting samples: (**a**) 0%; (**b**) 17.46%; (**c**) 38.19%; (**d**) 58.16%.

The upsetting experiment of 18Ni(250) maraging steel was carried out and the samples of different deformation amounts were obtained. The experimental results are shown in Figure 6. The compression amounts are 17.46%, 38.19% and 58.16%, and the sample heights after deformation are 103.18 mm, 77.26 mm and 52.3 mm, respectively. After deformation, bulging occurred on the side of the samples, and there were no cracks on the surface.

### 3.3. Microstructure Evolution of 18Ni(250) Steel after Deformation

**Figure 7:**
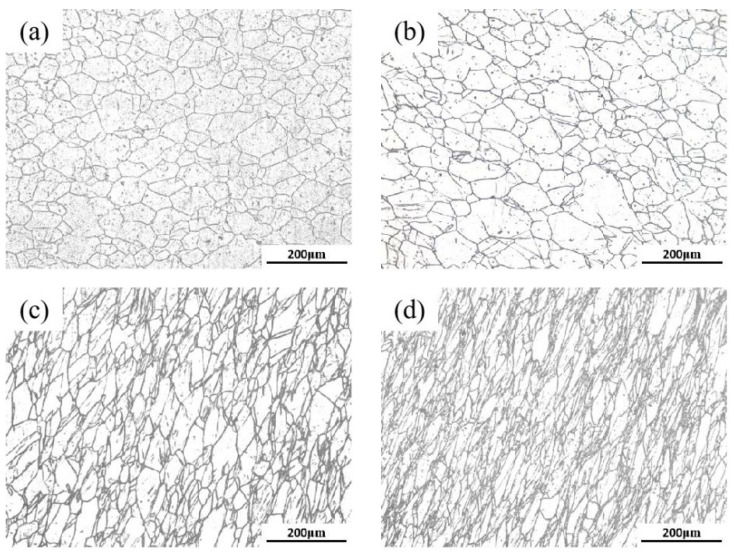
The metallographic photographs of C250 after deformation: (**a**) 15%; (**b**) 30%; (**c**) 45%; (**d**) 60%.

The metallographic photographs of 18Ni(250) steel with different deformation amounts are shown in Figure 7. The grain size was calculated by metallographic photographs, and the calculation results are shown in Table 1. The unforged 18Ni(250) steel has coarse grains with an average size of 327 μm [40]. After the deformation amount of 15%, the grain size was significantly reduced compared to the original material, a large number of fine grains appeared, and the average grain size was reduced to 30.7 μm. It can be found by comparing the calculation results that the greater the deformation amount, the smaller the grain size.

**Figure 8:**
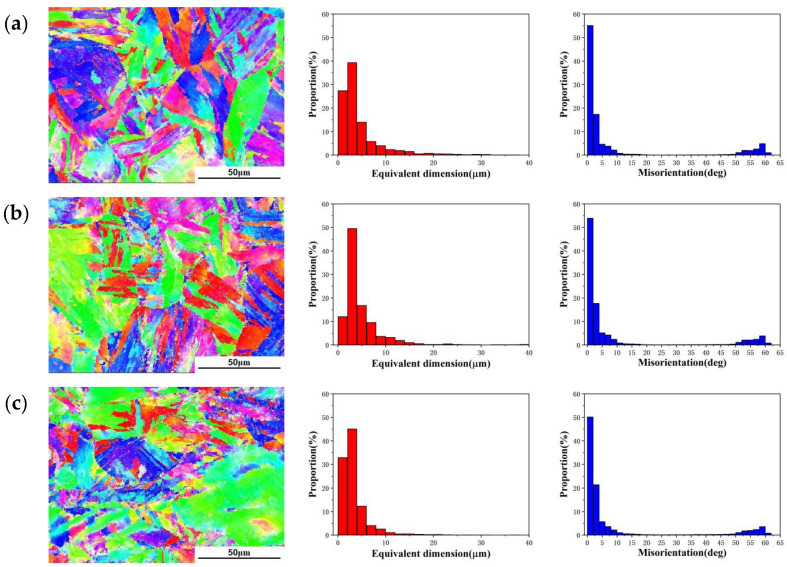

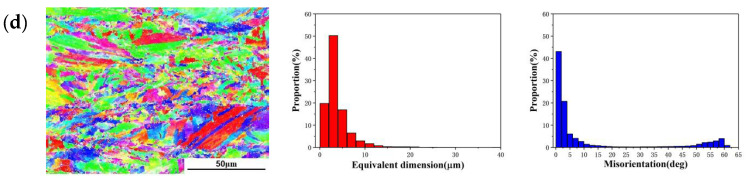
The distribution and dimensions of the martensite lath and the distributions of grain boundary misorientation after deformation: (**a**) 15%; (**b**) 30%; (**c**) 45%; (**d**) 60%.

Since it is difficult to observe the substructure of martensite by optical microscope, the microstructures of martensite were characterized by EBSD, and the distributions of martensite laths with different deformation amounts are shown in Figure 8. The average size of the martensite lath was calculated by EBSD photographs, and the calculation results are shown in Table 1. As the deformation amount increases, the grain size and the average size of the martensite lath gradually decreases. It can be observed from Figure 8a,b that when the deformation amount is small, the arrangement of martensite lath is regular. The size of martensite lath is distributed between 1 and 36 μm, and most of the size of martensite lath is less than 15 μm, accounting for about 92.5%. When the deformation amount is 60%, the size of martensite lath is significantly reduced, most of the lath size is less than 10 μm, and the proportion of martensite lath less than 10 μm is about 98.2%, as shown in Figure 8c,d. This is because a large number of uneven deformed microstructures are generated in the 18Ni(250) maraging steel during deformation, and there are a large number of dislocations and internal defects, which causes recrystallization during the forging process. When the deformation amount is large, large plastic deformation occurs inside the metal, and the stored energy increased by strain provides the driving force for recrystallization. Due to the high distortion energy, fine grains are obtained after recrystallization. The high distortion energy can increase the nucleation rate of martensite transformation and promote the refinement of martensite laths. The plastic deformation has a restraining effect on the growth of martensite and has an indirect effect its refining [41]. There are higher densities of dislocation tangle in recrystallized grains, which prevents the growth of martensite lath, so the size of the martensite lath is reduced [42].

The distributions of grain boundary misorientation of 18Ni(250) steel under different forging conditions are shown in Figure 8. As the deformation amount increases, the proportion of low-angle boundaries less than 10° gradually decreases, from 83.3% to 76.65%, and the proportion of high-angle boundaries of 50° to 60° gradually increases. This means that as the deformation amount increases, the low-angle boundaries gradually transform into high-angle boundaries. Because a large number of dislocations are generated inside the grains during the deformation process, the motion of the slip system in different regions is different, and the sub boundary is gradually formed. The sub boundary in the austenite is the low-angle boundary; recrystallization that occurs at this position gradually transforms the low-angle boundaries into the high-angle boundaries [40].

### 3.4. Microstructure Evolution of 18Ni(250) Steel after Heat Treatment

**Figure 9:**
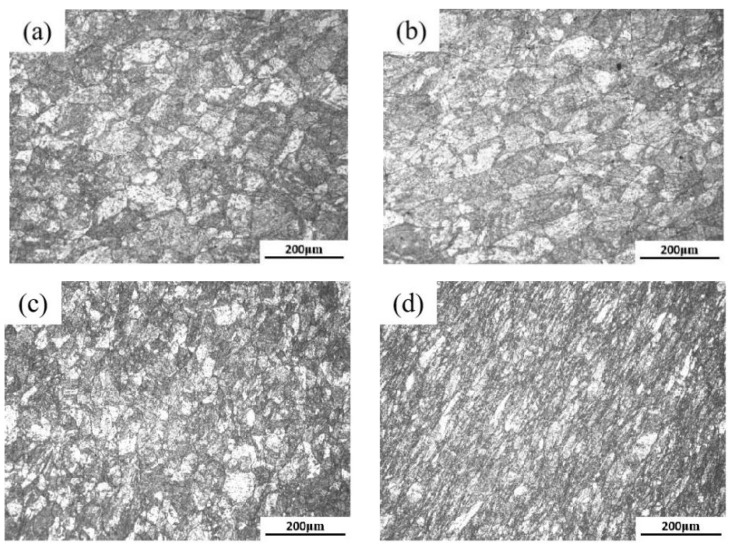
The metallographic photographs of C250 after heat treatment: (**a**) 15%; (**b**) 30%; (**c**) 45%; (**d**) 60%.

The metallographic photographs of 18Ni(250) steel with different deformation amounts are shown in Figure 9. The grain size was calculated by metallographic photographs, and the calculation results are shown in Table 1. After heat treatment, nanoscale precipitates such as Ni_3_Ti, Fe_2_Mo and Fe_7_Mo_6_ are precipitated in the maraging steel, which are uniformly and dispersed distributed on the dislocation lines and the martensite lath boundaries, thus affecting the clarity of the microstructure [5,7].

**Figure 10:**
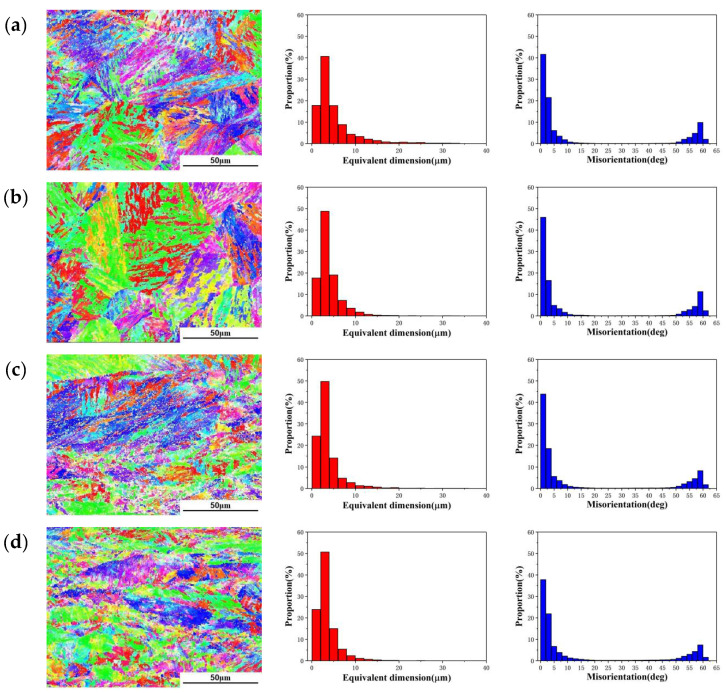
The distribution and dimensions of the martensite lath and the distributions of grain boundary misorientation after heat treatment: (**a**) 15%; (**b**) 30%; (**c**) 45%; (**d**) 60%.

The distributions of martensite laths with different deformation amounts after heat treatment are shown in Figure 10, and the average sizes of the martensite lath are shown in Table 1. The change trend of the size of grain and martensite lath with deformation amount after heat treatment is different from that after deformation. The average size of the martensite lath after heat treatment changes little with the deformation amount, but as the deformation amount increases, the grain size still decreases. The size of martensite lath is distributed between 1 and 20 μm, and most of the size of the martensite lath is less than 10 μm, accounting for about 97.5%. Compared with the samples after deformation, the maximum size of the lath is reduced, and the proportion of the small-sized lath is increased. Generally, the cyclic phase transformation is required to refine the grains; solution and aging heat treatment do not have the effect of refining grains. This is because the transformation between martensite and austenite occurs during heat treatment, and the martensite phase is a kind of shear type transformation, so a large number of microscopic defects will be generated in the matrix, which increases the storage energy of the material. The storage energy and the distortion energy caused by plastic deformation leads to recrystallization, which increases the recrystallization fraction after heat treatment, and the grain size is reduced compared with that after deformation.

### 3.5. The Mechanical Properties of 18Ni(250) Steel after Forging and Heat Treatment

**Figure 11:**
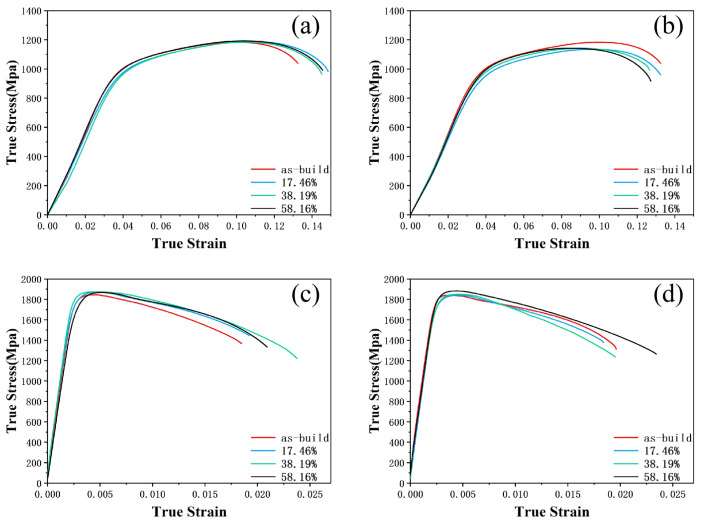
Tensile stress–strain curves of C250: (**a**) After deformation, CD; (**b**) After deformation, PD; (**c**) After heat treatment, CD; (**d**) After heat treatment, PD.

The tensile stress-strain curves of 18Ni(250) maraging steel under different deformation amounts and heat treatment conditions are shown in Figure 11. The compression direction is represented by CD, and the direction perpendicular to the compression direction is represented by PD. The 18Ni(250) maraging steel has higher ductility and lower strength before heat treatment, and the elongation is in the range of 13.8% to 16.3%, while the tensile strength is about 1065 MPa. When only thermal deformation is performed, as the deformation amount increases, the tensile strength in the compression direction increases by 9 MPa, that in the perpendicular to the compression direction increases by 19 MPa, and the elongation in both directions is similar after deformation. After heat treatment, the tensile strength of the material is increased to about 1860 MPa, and the elongation is reduced to about 2%.

## 4. Discussion

### 4.1. The Average Size of Martensite Lath Dependencies of Recrystallization and Grain Size

**Figure 12:**
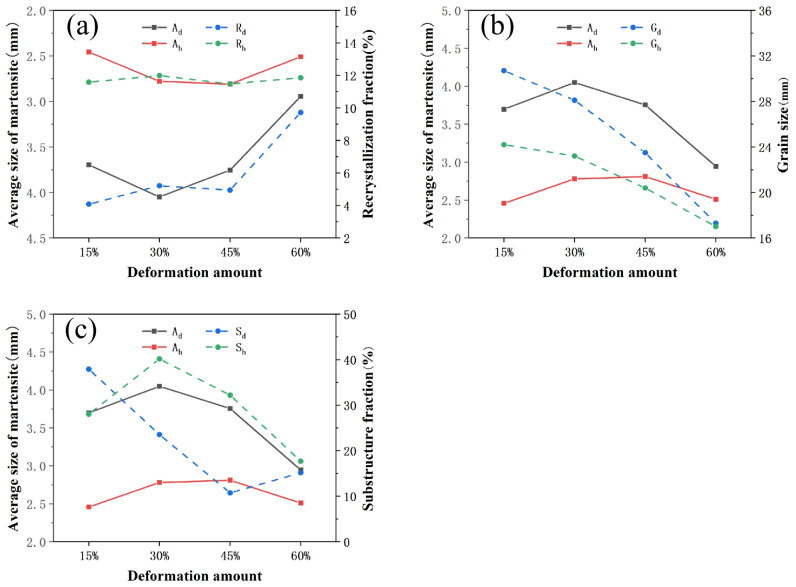
The average size of martensite lath dependencies of recrystallization fraction and grain size after forging and heat treatment: (**a**) Relationship with recrystallization fraction; (**b**) Relationship with grain size; (**c**) Relationship with substructure fraction.

The size of the martensite lath is affected by multiple factors such as the grain size and recrystallization. The recrystallization fraction, the grain size and the size of martensite lath after forging and heat treatment are shown in Figure 12. In the figure, A_d_ and A_h_ are the average size of the martensite lath after deformation and heat treatment respectively, R_d_ and R_h_ are the recrystallization fraction after deformation and heat treatment, respectively, G_d_ and G_h_ are the grain size after deformation and heat treatment, respectively, and S_d_ and S_h_ are the substructure fraction after deformation and heat treatment, respectively.

The recrystallization fraction after forging increases with the increase of deformation amount. The recrystallization fraction tends to be stable after heat treatment and has a significant increase compared to that after forging. The change of martensite lath size is opposite to the recrystallization fraction, and the change trend is approximately negatively correlated. The influence of recrystallization on martensite transformation is also related to the martensite substructure and grain size. The substructure of the martensite lath is generally high density of dislocations, the martensite substructure fraction can characterize the number of deformation dislocations. The larger the substructure fraction, the more deformation dislocations in the austenite. When there are few deformation dislocations, stress concentration of the parent phase is reduced and the martensite transformation is promoted. However, when a large number of deformation dislocations are generated in the austenite, work hardening will inhibit the martensite transformation [42]. The grain size affects the size of martensite laths in two aspects. The grain size decreases with the increase of deformation amount both after forging and after heat treatment. When the grain size is large, the grain boundary area is small and there are fewer martensite nucleation points. When the grains are refined, although the grain boundary area is increased, the yield strength of the parent phase is increased, which inhibits the martensite transformation [43]. It can be seen from the above analysis that with the increase of substructure fraction and grain size, the martensite transformation is first promoted and then inhibited.

In order to better analyze the effect of grain size and substructure on martensite transformation, the samples after the heat treatment can be taken as an example for analysis. The recrystallization fraction tends to be stable after the heat treatment, and the substructure fraction and grain size are different, which is convenient to comprehensively analyze the effect of grain size and recrystallization on the size of the martensite laths. The R_h_, S_h_, G_h_, and A_h_ of C250 after heat treatment are shown in Table 2.

When the deformation amount increases from 15% to 30%, the substructure fraction increases, the grain size decreases, and the martensite lath size increases. Although a large number of substructures inhibits the martensite transformation, the refined grain has a promotion effect on the martensite transformation, which is a cause for the growing of the martensite lath. When the deformation amount increases from 30% to 45%, the substructure fraction is decreased, and the effect of inhibiting the martensite transformation is weakened. However, the sizes of the martensite lath are almost equal. It is indicated that the martensite transformation has already been inhibited at this grain size. When the deformation amount increases from 15% to 60%, the martensite substructure fraction and the grain size decrease, while the martensite lath sizes of two deformation amounts are equal. It is indicated that there is both the promotion effect of the reduction of the substructure fraction and the inhibitory effect of the reduction of the grain size.

### 4.2. The Strengthening Mechanism of 18Ni(250) Maraging Steel

The yield strength $\sigma_{y}$ of maraging steel can be described by Equation (1) [44]:

(1) $$\sigma_{y}=\sigma_{0}+\sigma_{p}+\sigma_{s}+\sigma_{\rho }+k_{\mathrm{HP}}d^{-1/2}$$

In this equation: $\sigma_{0}$ is the frictional stress of pure iron, $\sigma_{p}$ is the precipitation hardening, $\sigma_{s}$ is the solid solution hardening, $\sigma_{\rho }$ is the hardening of dislocations in the martensite laths and the low-angle boundaries, and $k_{\mathrm{HP}}d^{-1/2}$ is the grain boundary strengthening ($k_{\mathrm{HP}}$ is Hall-Petch slope, d is the effective grain size, it is generally the size of martensite lath instead of grain size in maraging steel).

It can be seen from the formula that the strength of 18Ni(250) steel is affected by multiple strengthening mechanisms. After thermal compression, the size of the grain and the martensite lath and the ratio of low-angle boundaries decrease with the increase of the deformation amount. Therefore, only considering the deformation amount, the tensile strength is inversely proportional to the size of the martensite lath; the strength of 18Ni(250) steel is affected by the size of the martensite lath, but the effect is small. The interfacial energy of the low-angle boundaries is low, which hinders the dislocation slip and improves the strength of the 18Ni(250) maraging steel. With the increase of the deformation amount, the low-angle boundaries decrease, but the tensile strength increases. Consequently, dislocation strengthening is not the main strengthening mechanism of 18Ni(250) maraging steel. After heat treatment, as the deformation amount increases, the grain size of the 18Ni(250) maraging steel decreases, while the size of the martensite lath and the ratio of the low-angle grain boundaries remain basically unchanged. After heat treatment of solution and aging, the tensile strength is increased by about 800 MPa; this is due to the precipitation of nanoscale intermetallic compounds such as Ni_3_Ti, Fe_2_Mo and Fe_7_Mo_6_, which play a role in dispersion strengthening, and significantly affect the tensile strength and elongation of 18Ni(250) steel [5]. Precipitation strengthening and solution strengthening are the most important strengthening mechanisms of maraging steel.

**Figure 13:**
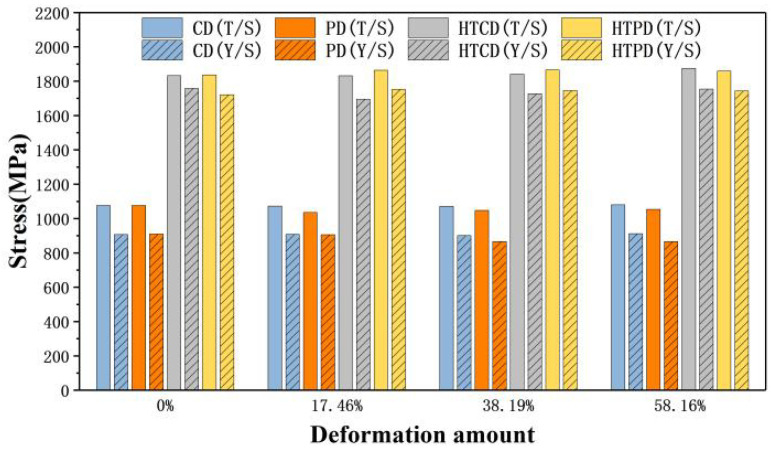
The tensile strength and yield strength of C250 after deformation and heat treatment.

**Figure 14:**
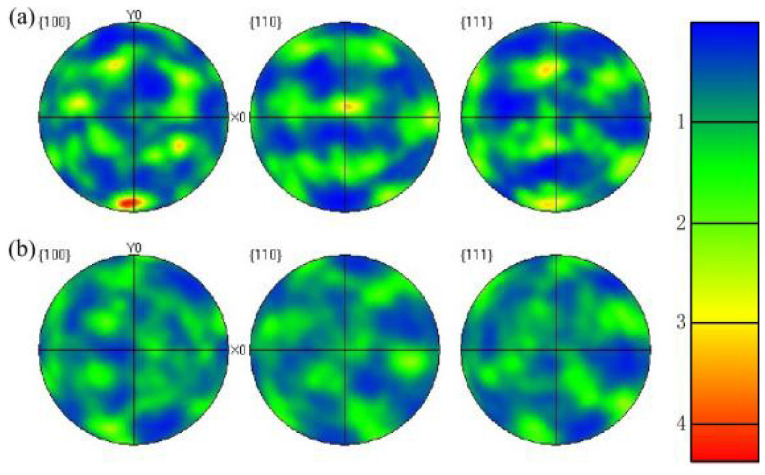
EBSD pole figures of C250: (**a**) After deformation; (**b**) After heat treatment.

The tensile strength and yield strength of 18Ni(250) steel under different deformation amounts and heat treatment conditions are shown in Figure 13. The mechanical properties of the original material in the compression direction and perpendicular to the compression direction are similar, without showing obvious anisotropy. After deformation, the tensile strength in the compression direction is about 1075 MPa, and the tensile strength perpendicular to the compression direction is about 1045 MPa, the tensile strength in the compression direction is slightly higher than that that is perpendicular to the compression direction. However, the tensile strength in both directions is about 1850MPa after heat treatment, which does not show anisotropy. This is because after compression deformation the fiber texture perpendicular to the compression direction will be generated inside the material, as shown in Figure 14a, which results in the anisotropy. After solution and aging heat treatment, the 18Ni(250) maraging steel is recrystallized and the texture significantly weakened, as shown in Figure 14b, so that it does not show obvious anisotropy after heat treatment.

The combination of plastic deformation and aging treatment can refine the martensite and increase the dislocation density. This method can accelerate the precipitation process of intermetallic compounds and make the precipitation phase more uniformly dispersed, which also has a great effect on eliminating anisotropy [31]. Generally, the grains of 18Ni(250) maraging steel are refined by cyclic phase transformation. The cyclic phase transformation requires multiple cool-heat cycles and takes a long time. The result of research shows that the distortion energy is stored inside the material after forging, and recrystallization can also occur during heat treatment, so that grain refinement can also be achieved, and the internal defects of the original material can be better eliminated. After heat treatment, the anisotropy caused by forging can be eliminated, which would not affect the properties of the material. Therefore, thermal deformation is better at grain refinement and internal defect elimination compared with cyclic phase transformation.

## 5. Conclusions

Through the study of the microstructural and mechanical properties of 18Ni(250) steel under different deformation amount and heat treatment conditions, the results can be outlined as follows:

1. The change of the martensite lath size is opposite to the recrystallization fraction, and the change trend is approximately negatively correlated. The influence of recrystallization on martensite transformation is also related to the martensite substructure and grain size, since with the increase of martensite substructure fraction and grain size, the martensite transformation is first promoted and then inhibited.
2. The strength of 18Ni(250) steel is affected by multiple strengthening mechanisms. Grain refinement strengthening is the main strengthening mechanism during forging, precipitation strengthening and solution strengthening are the most important strengthening mechanisms during heat treatment.
3. After solution and aging heat treatment, the 18Ni(250) maraging steel is recrystallized and the texture generated by uniaxial compression deformation is significantly weakened, so that it does not show obvious anisotropy after heat treatment.
4. Forging combined with heat treatment can refine grains and the martensite lath, and the internal defects of the original material can be better eliminated; thermal deformation can better play the role of grain refinement compared with cyclic phase transformation, which can improve the plasticity of 18Ni(250) maraging steel.
5. The strengthening mechanism of forging and heat treatment are investigated, which can be applied to the design of forgings with high performance requirements in the future, which is helpful for developing a robotic free forging process and improving the intelligent manufacturing level of key aviation forgings of maraging steel.

## Figures and Tables

**Table 1:** The size of grain and martensite lath of C250 after deformation and heat treatment.

Deformation Amount	The Grain Size		The Average Size of the Martensite Lath	
	Deformation	Heat Treatment	Deformation	Heat Treatment
15%	30.7 μm	24.2 μm	3.70 μm	2.46 μm
30%	28.1 μm	23.2 μm	4.05 μm	2.78 μm
45%	23.5 μm	20.4 μm	3.757 μm	2.81 μm
60%	17.3 μm	17.0 μm	2.947 μm	2.51 μm

**Table 2:** The R_h_, S_h_, G_h_, A_h_ of C250 after heat treatment.

No.	Deformation Amount	R_h_/%	S_h_/%	G_h_/μm	A_h_/μm
1	15%	11.5883	28.0148	30.7	2.46
2	30%	11.9901	40.1456	28.1	2.78
3	45%	11.4710	32.1939	23.5	2.81
4	60%	11.8554	17.6714	17.3	2.51

## Data Availability

Data sharing not applicable.
